# Targeting the premetastatic niche: epigenetic therapies in the spotlight

**DOI:** 10.1038/s41392-020-0165-3

**Published:** 2020-05-11

**Authors:** Vivien Low, John Blenis, Ana P. Gomes

**Affiliations:** 1 Meyer Cancer Center, Weill Cornell Medicine, New York, NY USA; 2 Department of Pharmacology, Weill Cornell Medicine, New York, NY USA

**Keywords:** Metastasis, Molecular medicine

The treatment of metastatic cancers remains a challenge in the clinic, with recurrences persisting at high rates even after surgical resection of primary tumors. Beyond the perseverance of circulating tumor cells after resection, this phenomenon is abetted by the formation of the premetastatic niche (PMN), a distant environment ripe for the colonization of new metastases, which is conditioned by factors and extracellular vesicles secreted by the primary tumor. In their recent paper, Lu et al. demonstrate how such PMNs in the lung can be disrupted by low-dose adjuvant epigenetic therapy, and propose a novel therapeutic avenue to supplement tumor resection.^1^

The evolution of the PMN necessitates the effects of systemic tumor-secreted factors and vesicles upon an organ-specific microenvironment conducive to the outgrowth of circulating tumor cells,^2^ and is suggested to follow a sequential paradigm. As the tumor progresses, tumor cells secrete angiomodulatory factors such as vascular endothelial growth factor A (VEGFA) and angiopoietin-like4 (ANGPTL4), as well as cytokines including chemokine C–C motif ligand 2 (CCL2). Increased vascular permeability precedes the alteration of resident cells in the pre-stromal environment as well as the recruitment of non-resident cells, such as bone marrow-derived cells (BMDCs), which then primes the PMN for colonization. Lu et al. explore how the trafficking of a subset of these BMDCs, called myeloid-derived suppressor cells (MDSCs), can be effectively disrupted by low-dose adjuvant epigenetic therapy (AET), a combination of DNA methyltransferase inhibitor 5-azacytidine and histone deacetylase inhibitor entinostat.

MDSCs are a heterogenous group of immature immune cells established to play a key role in cancer through their suppressive activity on immune effector cell function. High MDSC infiltration in cancer tissues has been associated with poor clinical outcomes and therapeutic resistance.^3^ Using three syngeneic mouse models of aggressive lung metastasis: Lewis lung carcinoma (LLC), HNM007 oesophageal squamous cell carcinoma, and 4T1 mammary cancer, Lu and colleagues show that MDSCs accumulate in the lung PMN and contribute to metastasis formation after primary tumor resection. While no tumor-cell infiltration was detectable until day 6 after resection of the primary tumor, MDSCs were already enriched in the lung at day 0 of resection. Depletion of MDSCs was sufficient to prolong metastasis-free and overall survival, while intravenous injection of MDSCs from day 3-LLC mice increased the rate of metastasis and decreased survival, suggesting a functional role of MDSCs in the PMN. MDSCs can be largely classified by two major subsets: monocytic MDSCs have a monocyte-like morphology and phenotype and are characterized by high plasticity, while polymorphonuclear MDSCs have a morphology and phenotype similar to neutrophils. Lu et al. showed that although both subtypes were enriched in the PMN, transfer of specifically monocytic, but not polymorphonuclear, MDSCs resulted in accelerated metastasis formation.

Epigenetic therapy using a combination of 5-azacytidine and entinostat has been studied and met with limited success in certain metastatic cancers, including colorectal and breast cancer,^4,5^ despite being generally well tolerated. Promising observations in a small trial of NSCLC patients led Lu and colleagues to explore this combination as an adjuvant treatment to early primary tumor resection. They found that low-dose AET had a negligible effect on proliferation, viability and apoptosis, had no effect on tumor growth and did not cause weight loss in immune-compromised mice with tumors. It did, however, produce a significant decrease in the presence of MDSCs as well as niche-promoting molecules in the lungs of the three syngeneic mouse models by impeding the migration of MDSCs into the PMN. Lu et al. identified CCR2, a chemokine receptor essential for monocytic cell migration, that was downregulated by AET in monocytic MDSCs in the bone marrow and lung. In vitro, monocytic MDSCs also showed reduced migration upon AET in transwell assays induced by CCL2, the CCR2 ligand. When authors knocked out CCR2 in mice, monocytic MDSC levels were negligible in the premetastatic lung compared to wildtype mice, and disease-free and overall survival was increased. The authors proposed that AET may affect CCR2 expression through downregulation of the NF-κβ signaling pathway, which is downregulated in AET-treated monocytic MDSCs, and whose inhibition decreases expression of CCR2 in monocytic MDSCs in vivo. In a similar set of experiments, authors found that AET decreased the trafficking and migration of polymorphonuclear MDSCs to the lung through downregulation of CXCR2, implicating CCR2 and CXCR2 as the regulators of monocytic and polymorphonuclear MDSC trafficking, respectively, to the PMN.

**Fig. 1:**
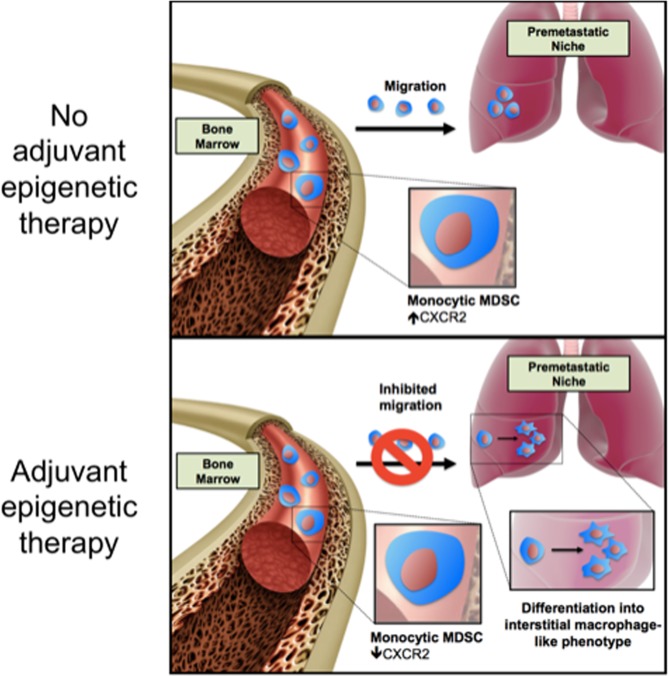
Adjuvant epigenetic therapy (AET) disrupts the premetastatic niche (PMN) by inhibiting trafficking of monocytic myeloid-derived suppressor cells (MDSCs) and promoting their differentiation. Monocytic MDSCs from the bone marrow accumulate in the lung PMN before colonization by circulating tumor cells. AET results in a downregulation of CCR2, a key regulator of monocytic cell migration, which inhibits the migration of monocytic MDSCs into the lung. Additionally, AET promotes the differentiation of the monocytic MDSCs that manage to migrate to the PMN into a more interstitial macrophage-like phenotype, antagonizing their immunosuppressive and pro-tumor effects

Lu et al. found that in addition to inhibiting migration, AET also decreased levels of monocytic MDSCs in the PMN by promoting their differentiation into an interstitial macrophage-like phenotype, counteracting their function in supporting the PMN (Fig. 1a). Through these two mechanisms, Lu and colleagues successfully used AET to reduce pulmonary metastasis and prolong survival in three mouse models, and furthermore discovered a synergistic effect of AET with a CCR2 antagonist. Intriguingly, depletion of CD4 and CD8 T cells did not change the effects of low-dose AET regarding metastasis formation or survival, suggesting that this phenotype is T cell independent. As MDSCs are known to be suppressors of T cell activity, it will fascinating to follow how further research reveals the ways in which MDSCs may alternatively exert their anti-metastatic functions.

Due to the complexity and prominence of epigenetic events in cancer pathogenesis, there are many prospects for conceivable epigenome-targeted therapies. In fact, 5-azacytidine, one of the two drugs used in Lu and colleague’s AET, has been FDA-approved for the treatment of chronic myelomonocytic leukemia. Importantly, the historical advantage of using nucleoside analogs such as 5-azacytidine is their preference for newly synthesized DNA, thus their tendency to affect dividing cancer cells. In contrast, Lu et al. propose AET as a potential strategy not for its ability to target tumor cells themselves, but rather the cells that contribute to the delineation of a pulmonary PMN and enable cancer recurrence. It remains to be seen whether PMNs in other organs and tissues could be targeted in the same manner. All in all, Lu and colleagues’ findings create the framework for a potential novel application of low-dose AET in combination, with CCR2 antagonists, particularly in in the context of an absent primary tumor.
